# Delay of Initial Feeding of Zebrafish Larvae Until 8 Days Postfertilization Has No Impact on Survival or Growth Through the Juvenile Stage

**DOI:** 10.1089/zeb.2018.1579

**Published:** 2018-10-03

**Authors:** Rafael E. Hernandez, Louie Galitan, James Cameron, Nicola Goodwin, Lalita Ramakrishnan

**Affiliations:** ^1^ Department of Pediatrics, University of Washington, Seattle, Washington.; ^2^ Center for Global Infectious Diseases Research, Seattle Children's Research Institute, Seattle, Washington.; ^3^ Department of Microbiology, University of Washington, Seattle, Washington.; ^4^ Molecular Immunity Unit, MRC Laboratory of Molecular Biology, Department of Medicine, University of Cambridge, Cambridge, United Kingdom.; ^5^ Department of Medicine, University of Washington, Seattle, Washington.

**Keywords:** feeding, growth, survival, animal regulation

## Abstract

The use of early-stage zebrafish for biomedical research spans early organogenesis to free-swimming larva. A key benefit of this model organism is that repeated assessments spanning several days can be performed of individual larvae within a single experiment, often in conjunction with administered drugs. However, the initiation of feeding, typically at 5 days postfertilization (dpf), can make serial assessments challenging. Therefore, delayed feeding would increase the utility of the model. To ask whether feeding could be delayed without adversely affecting larval growth and development up to 39 dpf, we systematically raised zebrafish and introduced feeding at 5 dpf or delayed initial feeding up to 9 dpf. We assessed survival into the juvenile stage (39 dpf) and anterior-posterior length at this age as proxies for growth and development. Delaying feeding initiation up to 8 dpf did not decrease baseline survival of greater than 90%; survival decreased to 66% only when delayed to 9 dpf. Larval length was no different under any of these conditions. Our findings define 9 dpf as the critical age before which larval zebrafish must be fed when raising to 39 dpf. The option to delay feeding to 8 dpf will broaden experimental applications for the zebrafish larval model.

## Introduction

Experimentation with zebrafish often requires manipulation and observation after larval hatching and through the first several days when larvae are expected to be free swimming and may be able to initiate feeding. Zebrafish larvae inflate their swim bladder and become free swimming between ∼96 and 144 h postfertilization (hpf).^1^ The age of initial feeding when rearing zebrafish varies but usually coincides with this transition to free swimming, with international zebrafish resource facilities initiating feeding between 5 and 6 days postfertilization (dpf).^2,3^ However, the age range at which zebrafish larvae must initiate feeding for normal survival and maturation into adulthood has not been systematically determined.

Zebrafish larvae are employed in a broad range of research and are useful models for multiple lines of scientific inquiry, including the neurologic basis of behavior, regeneration, toxicology, and host–pathogen interactions among others.^4^ High throughput and/or repeat observation of individual larval outcomes over time can be significantly limited by the need to initiate feeding. There is no way to mark individual larva and food added to individual wells in a multiwell screening plate can breakdown, generating ammonia, fouling water quality, and potentially interfering with planned observations. Therefore, we sought to determine the age by which feeding must be initiated to prevent detrimental effects on growth and survival of juveniles up to 39 dpf.

## Materials and Methods

Zebrafish husbandry and experiments were conducted according to guidelines from the US NIH (approved by the University of Washington Institutional Animal Care and Use Committee) and the U.K. Home Office.

Adult broodstock were maintained on a recirculating system with the following system water parameters: conductivity 800–1000 μS/cm (Instant Ocean), pH 7.0–7.5 (buffered with sodium bicarbonate), and temperature 28°C. Wild-type AB zebrafish embryos were produced by group matings of 2 male and 2–3 female broodstock zebrafish. Embryos were rinsed for 1 min in dilute bleach (0.004% sodium hypochlorite) in fish system water, then in clean fish system water, repeating for a total of three bleach rinses.

**FIG. 1.**
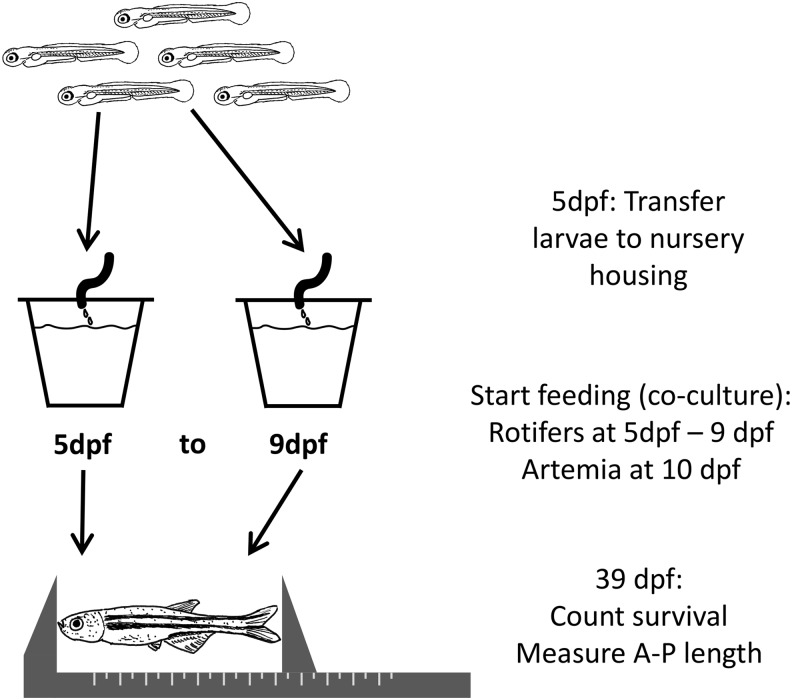
Feeding timeline and rearing strategy. Larvae at 5 dpf were transferred to a recirculating housing tank system at relatively high salinity (conductivity 8000–9000 μS) used exclusively for larval fish. Feeding was initiated at age 5 to 9 dpf by coculture with live salt-water rotifers. Artemia nauplii were added at 10 dpf and later supplemented with commercial feed. At 39 dpf, fraction of fish surviving was assessed, and length of individual fish was measured with calipers. dpf, days postfertilization.

Fertile embryos were partitioned into 100 embryos per 10 cm petri dish, with ∼20 mL of sterile filtered fish system water, and held at 28°C with one water change at 1 dpf. For delayed feeding trials (Fig. 1) larvae at 5 dpf were transferred to housing tanks with 400 μM screens at a density of 22 larva per 3 L tank. These tanks were placed on a recirculating system, with a relatively high salinity of 4 ppt (conductivity 8000–9000 μS), used exclusively for larval fish. Tanks were initially filled to ∼2.5 cm depth and initial flow in the tanks was set at very slow drip (2–3 mL/min).

Zebrafish larvae were fed in a polyculture tank system similar to previously described.^5^ Stock rotifers (saltwater L strain; Reed Mariculture) were cultured in salt water (salinity of 8 ppt or 13,000 μS) with Instant Algae Nano 3600 Nannochloropsis (Reed Mariculture) and maintained at densities between 50 and 100/ml. Feeding of zebrafish was initiated at 5 dpf, or delayed per treatment group to the specified age, by adding rotifers, rinsed and suspended to a density of ∼200 rotifers/mL, with a small amount of fresh algae paste. Larvae were then fed exclusively on rotifers (30–50 mL daily) until age 10 dpf, when larvae were freely swimming throughout the water column. Artemia nauplii, freshly hatched from decapsulated San Francisco Strain Brine Shrimp Eggs (brineshripdirect.com), were introduced, starting around 50 to 100 nauplii per tank and increasing in numbers to maintain excess nauplii.

At age 15 dpf larvae were fully weaned off rotifers onto Artemia and formulated diets (BioVita Starter Mash, sifted for particles <0.5 mm, purchased from Bio-Oregon). Adult broodfish were fed formulated diets (#0 BioVita Starter, Bio-Oregon) with Artemia nauplii supplements twice per day.

At 39 dpf, the approximate age that juvenile fish would have been transferred to the adult circulating system with lower salinity, the final count of surviving juvenile fish per tank was determined. Fish were given terminal anesthesia with sodium bicarbonate buffered tricaine (400 mg/L). Final anterior-posterior larval length was measured to the nearest mm with calipers. Images of the juvenile fish were captured with a handheld digital camera on a plastic dish with a reference mark used to determine scale. In Adobe Photoshop CC a scale bar was added, images were adjusted to the same scale, rotated, cropped, and transformed to grayscale.

All data presented from delayed feeding trials are pooled data from two tanks of fish per time point, *n* = 22 per tank, except photographs of juvenile fish that represent one of two tanks. Statistical analysis was performed with GraphPad Prism 6. The fraction of fish surviving and associated 95% confidence intervals were calculated for each group by the Clopper-Pearson method. Chi square analysis was used to assess variation in survival across all groups, with individual pairwise comparisons between groups made by Fisher's exact test. One-way ANOVA was used to determine whether there were any differences in juvenile fish length at 39 dpf, with a post-test for linear trend across the groups and Holm-Sidak post-test to identify significant differences between groups. Specific analyses are noted in figure legends.

Data for comparative survival of larval fish raised by routine feeding with paramecium were collected at Cambridge University during a 24 month period. Larval AB zebrafish were transferred to tanks, with 25 larvae per tank and provided 100 mL of paramecium per day from 5 to 15 dpf. During this time period no flow of water was provided to the tanks. From 16 to 30 dpf larva were provided system water of a slow drip flow from a recirculating system with routine salinity (550–650 μS). From 16 dpf Artemia nauplii were fed twice per day and fine prepared dry food three times per day. The fraction of larvae surviving nursery rearing was assessed at 30 dpf. The number of fish in each group is indicated in the figure legend.

## Results

**FIG. 2.**
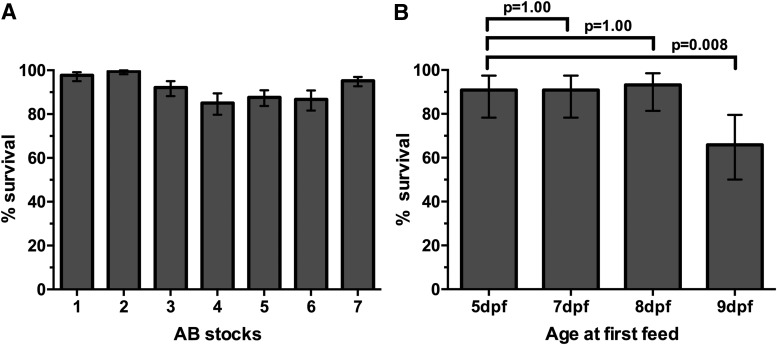
Effect of age of first feeding on zebrafish survival. **(A)** Routine survival of wild-type zebrafish larvae with first feeding at 5 dpf raised on an initial diet of paramecia. Columns represent survival rates of individual stocks of AB wild-type larvae raised to 30 dpf in the nursery (AB Stock #1, *n* = 261; #2, *n* = 400; #3, *n* = 266; #4, *n* = 225; #5, *n* = 347; #6, *n* = 233; #7, *n* = 435). **(B)** Proportion of fish surviving to 39 dpf in rotifer co-culture system, depending on larval age at first feed. Data are pooled from two tanks (*n* = 22 per tank, 44 total) per group. The portion surviving was significantly lower in the 9 dpf group compared with the 5 dpf group (*p* values represent pairwise comparisons by Fisher's exact test). Error bars in **(A)** and **(B)** represent 95% CI.

We pooled AB embryos from group spawns and transferred 5 dpf larvae to housing tanks on a recirculating system. As detailed in the Methods and Materials section, feeding practices vary between facilities and two commonly used ones include a coculture system with live saltwater rotifers (University of Washington facility) versus feeding the larvae paramecia (University of Cambridge). We confirmed that both feeding practices gave similar survival rates. Wild-type zebrafish stocks fed with paramecia ranged from 88% to 99.5%, with a median survival of 92.3% (Fig. 2A). Zebrafish on the rotifer coculture system routinely gave similar survival rates, as exemplified in the first column of Figure 2B.

The delayed feeding trials were initiated in the rotifer coculture system comparing survival after initiating feeding at 5 dpf to delaying feeding to between 6 and 10 dpf (Fig. 1).^5^ We initially performed a preliminary experiment, with 10 larvae per group, starting feeding between 5 to 10 dpf. All larvae in the group starting feeding at 10 dpf died within 2 days of initial feeding, without significant immediate mortality in the other groups (data not shown). To minimize unnecessary distress to additional animals, we dropped the 10 dpf feeding time point and compared feeding initiation at 5 to 9 dpf for further analysis. To reduce potential tank effects, two tanks of 22 larvae each were set up per time point on different days.

We observed significant variation in survival rates to juvenile stages between groups starting to feed at age 5 to 9 dpf (Fig. 2B, chi-square test, *p* = 0.0006). This variation appears entirely due to decreased survival in the 9 dpf group compared with the 5, 7, or 8 dpf groups (66% vs. 91%, 91%, and 93% respectively, Fig. 2B). Importantly, when feeding was initiated at 8 dpf, the rate of survival was virtually identical to the 5 dpf group (Fig. 2B).

**FIG. 3.**
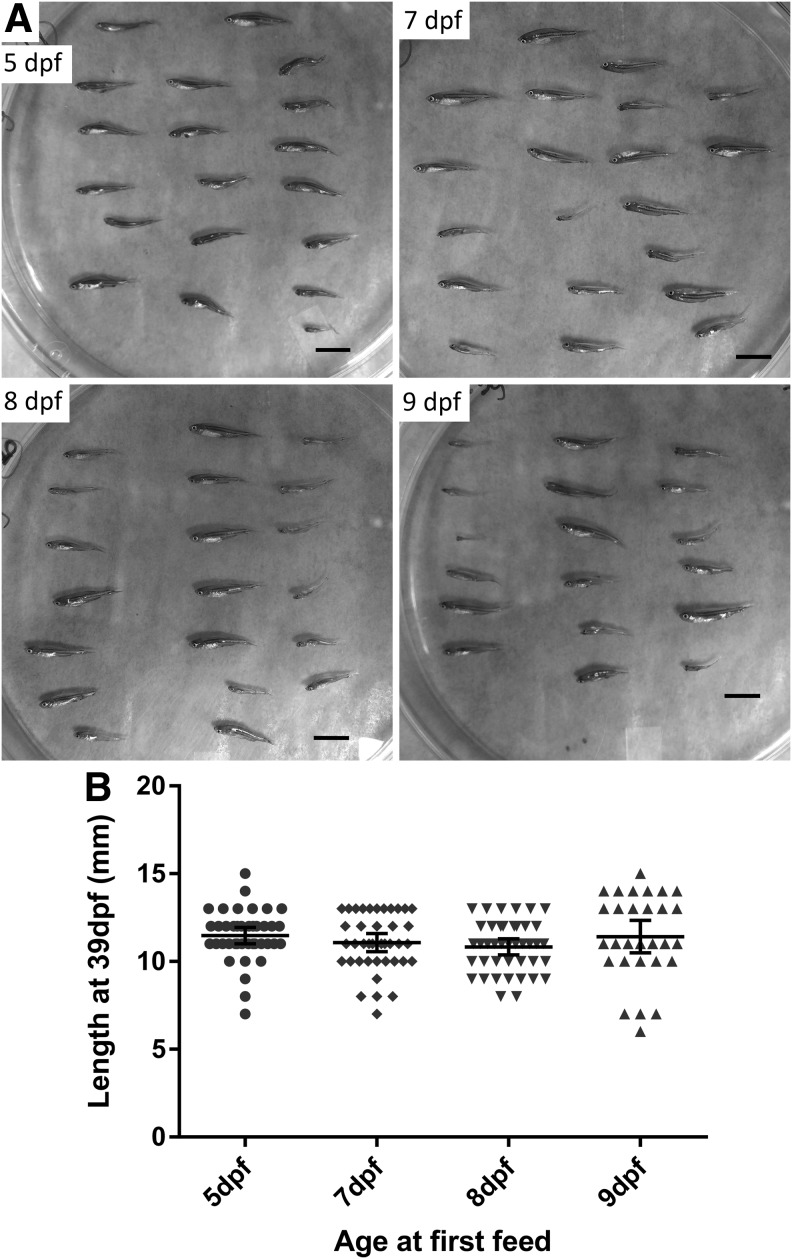
Effect of age of first feeding on zebrafish growth. **(A)** Representative images of all surviving juvenile zebrafish fish from a single tank that started feeding at indicated age. Scale bar represents 6.2 mm. **(B)** Length in mm of zebrafish surviving to 39 dpf, representing the same fish presented in Figure 2B. Lines and error bars represent mean and 95% CI of the mean. No significant differences were identified between 5 dpf and other groups by ANOVA and Holm-Sidak's multiple comparison test.

The lack of impaired survival due to delay of feeding to 8 dpf suggests that it did not cause irreparable harm in larvae. In addition to affecting survival into adulthood, severe larval malnutrition may be expected to impact linear growth. Visual examination of the juvenile fish showed that the majority of the fish across the groups initiating feeing at different ages appear similarly developed and sized at 39 dpf (Fig. 3A). We measured anterior-posterior lengths with calipers and found them to be similar across all feeding groups, with mean lengths of the fish in each feeding group within 1 mm of one another (Fig. 3B).

## Discussion

This work demonstrates that delaying the initiation of feeding to 8 dpf does not decrease larval survival and/or growth of zebrafish up to 39 dpf. We observed decreased survival only when feeding was delayed to 9 dpf, suggesting that there is a critical period between 8 to 9 dpf when zebrafish larvae have consumed the nutrients stored in their yolk so that catabolism is no longer sufficient to support growth and survival. We note that with initial larval feeding at 5 dpf our survival rate of 91% is very similar to reported rates of 93% when feeding salt water rotifers and Artemia in a coculture system.^5^ Survival is also similar to our observed rates in paramecia fed larvae.

Furthermore, while routine surveillance in our facility has intermittently identified coleps and microsporidia, we did not observe any significant illness or mortality due to these pathogens or other illness on routine health observations in fish subjected to delayed initial feeding. Therefore, we have no reason to believe our husbandry practices resulted in decreased survival that would mask any adverse effects of delayed initial feeding. Neither did delay in feeding produce any apparent increase in opportunistic infections.

These findings broaden the potential experimental scope and applications for the zebrafish larval model. Animal use ethic boards have generally regulated the use of zebrafish either at the time of hatching, ∼3 dpf,^6^ or at the time that larvae are expected to be free swimming and feeding, at 5 dpf.^7^ These cutoffs are obviously arbitrary. Our findings that larvae are not dependent on external food until 8 dpf to ensure growth and survival to juvenile stages warrant a reconsideration of the age at which zebrafish experimental use necessitates regulation.

Our data presented here are insufficient to support delaying feeding initiation beyond the current standard of 5 dpf when intending to raise larvae to adulthood. This work has created the foundation for future trials where larvae fed starting at 8 dpf are monitored into adulthood to monitor the effects of delayed feeding on sex bias, fecundity, and other parameters. If there are no detrimental effects, it will warrant consideration of regulatory amendments that allow delaying start of feeding to 8 dpf in both breeding and experimental contexts. In the meanwhile, on the basis of the current findings, we propose that for zebrafish being used within a study up to 39 dpf, regulatory requirements of feeding be delayed until 8 dpf. This will serve to broaden the use of this facile, versatile, and inexpensive animal model without significantly compromising animal welfare.
